# Gene expression profiling of the DNMT3A R882 mutation in acute leukemia

**DOI:** 10.3892/ol.2013.1347

**Published:** 2013-05-14

**Authors:** XIANGNAN HUANG, DAOXIN MA, WENHAO DONG, PENG LI, TING LU, NA HE, TIAN TIAN, NA LIU, YAHUI DU, CHUNYAN JI

**Affiliations:** Department of Hematology, Qilu Hospital of Shandong University, Jinan, Shandong 250012, P.R. China

**Keywords:** DNMT3A, R882 mutations, acute myeloid leukemia, acute lymphoblastic leukemia, gene expression

## Abstract

DNA methyltransferase 3A (DNMT3A) is one of two human *de novo* DNA methyltransferases essential for the regulation of gene expression. DNMT3A mutations and deletions have been previously observed in acute myeloid leukemia (AML), myelodysplastic sydromes and myeloproliferative neoplasms. However, the involvement of DNMT3A in acute lymphoblastic leukemia (ALL) has rarely been reported. In the present study, PCR and direct sequencing was performed to analyze mutations of DNMT3A amino acid residue 882 in 99 acute leukemia patients, including 57 AML patients, 41 ALL patients and a single biphenotypic acute leukemia (BAL) patient. DNMT3A expression was detected in mono-nuclear cells of the bone marrow in these patients and in normal individuals using real-time quantitative polymerase chain reaction, and 17.5% (10/57) of AML patients were found to exhibit DNMT3A mutations. Four missense mutations were observed in the DNMT3A-mutated AML patients, including R882 mutations and a novel single nucleotide polymorphism resulting in the M880V amino acid substitution. However, the ALL and BAL patients were not found to exhibit DNMT3A mutations. The DNMT3A expression levels in the AML patients were significantly higher compared with those of the ALL patients or normal controls. The reduced expression levels of DNMT3A were associated with a significantly lower complete remission rate in the AML patients. However, in the ALL patients, no statistical significance was identified. The results of the present study indicate that DNMT3A may play varying roles in the regulation of DNA methylation in AML and ALL.

## Introduction

Acute leukemia (AL) consists of a group of heterogeneous malignancies in which immature and dysfunctional hematopoietic progenitors proliferate and accumulate in the bone marrow. DNA methylation plays a key role in the pathophysiology of acute myeloid leukemia (AML) (1) and acute lymphoblastic leukemia (ALL) (2,3). DNA methyltransferase 3A (DNMT3A) is one of two human *de novo* DNA methyltransferases essential for regulating gene expression during cellular development and differentiation (4). DNMT3A mutations and deletions have been analyzed in AML (5), chronic myeloid leukemia (CML), chronic myelomonocytic leukemia (CMML), myelodysplastic syndrome (MDS), lymphoma and myeloproliferative neoplasms (MPNs) (6–11). The frequency of the mutations in patients with different diseases varies between 0 (0/81, CML patients in blast crisis) and 22.1% (62/281, AML patients). The most common mutation has been identified at the site of amino acid residue R882.

DNMT3A mutations have been found to be enriched in the M4 (32.8%) and M5 subtypes (57.1%), according to the French-American-British (FAB) classification system (12). The DNMT3A expression levels in patients with DNMT3A mutations were observed to be marginally lower than those without mutations. In addition, DNMT3A was also found to be expressed in normal human CD34^+^ bone marrow cells and its expression decreased with terminal myeloid differentiation (5). However, to date, the differences in the DNMT3A expression levels in various subtypes of AML, according to FAB classification, have not been determined. In a previous study, the results of a multivariate analysis indicated that DNMT3A mutations represented independent predictive factors of poor prognosis, including a reduced overall survival (OS) and complete remission (CR) rate (7). Qiao *et al* (13) reported that the CR rate in acute leukemia (AL) patients that were identified to positively express all the DNMT genes was significantly higher than that of patients with partially positive or negative expression, indicating that DNMT3A mutations and expression may be associated with the pathogenesis and prognosis of AL.

ALL is a heterogeneous malignancy caused by the clonal proliferation of lymphocytes. The pathogenesis and causal cancer genes associated with AML and ALL differ (14). Unlike AML, extremely little is known about the mutation frequency of DNMT3A in ALL patients. Therefore, in the present study, 99 Chinese AL patients were screened for DNMT3A R882 mutations, with the aim of uncovering the frequency of the R882 mutations in ALL and the relationship between ALL and AML. In addition, DNMT3A expression levels were determined in these samples and normal controls to determine whether expression levels correlate with poor prognosis. The results demonstrate that the DNMT3A mutation status in AML is an important factor to consider for risk stratification of the disease.

## Materials and methods

### Patients, healthy subjects and bone marrow mononuclear cell (BMMC) and peripheral blood mononuclear cell (PBMC) collection

The study recruited 99 consecutive adult patients with AL [AML, ALL and biphenotypic acute leukemia (BAL)] newly-diagnosed at Qilu Hospital of Shandong University between August 2011 and November 2012. The diagnosis was made according to the FAB classification. For the clinical analysis, CR, partial remission (PR) and non-remission (NR) were defined according to the criteria of the International Working Group (15). Cytogenetic risk was determined in the AML patients following a method described previously (16). The characteristics of the patients at the time of sampling are presented in Tables I and II. The patients with AML were treated with standard induction chemotherapy (anthracycline and cytarabine). The patients with ALL were treated with standard induction chemotherapy (vincristine, daunorubicin, L-asparaginase and prednisone). In addition, a control group of 16 healthy donors was included. An assessment of the patient history and a physical examination were performed during the initial diagnosis. The corresponding laboratory tests were performed. BMMCs and PBMCs were obtained from 41 ALL patients (bone marrow or whole blood), 57 AML patients, one BAL patient and 16 control individuals (bone marrow) using density-gradient centrifugation with the Ficoll-Hypaque technique (Ficoll, Pharmacia LKB Biotechnology Inc., Piscataway, NY, USA). The samples were then stored at −80°C.

The present study was approved by the ethics committee of Qilu Hospital, Shandong University (Jinan, China). Written informed patient consent was obtained from all participants for the treatment and cryopreservation of BM and peripheral blood according to the Declaration of Helsinki.

### Genomic DNA isolation, PCR amplification and sequencing

Genomic DNA samples from bone marrow or whole blood of AML and ALL patients were extracted using the TIANamp genomic DNA kit [Tiangen Biotech (Beijing) Co., Ltd., Beijing, China] or the total DNA/RNA/protein extraction kit (Omega Bio-Tek, Inc., Norcross, GA, USA). A DNA fragment of 379 bp covering the R882 site in exon 23 of the DNMT3A gene was amplified using the S1000 thermal cycler (Bio-Rad, Hercules, CA, USA). Forward primer, 5′-TCC TGC TGT GTG GTT AGA CG-3′; and reverse primer: 5′-TAT TTC CGC CTC TGT GGT TT-3′. PCR was performed in a 25-*μ*l volume containing 30 ng DNA, 12.5 *μ*l PCR mastermix, 1 *μ*l forward primer, 1 *μ*l reverse primer and ddH_2_O. The PCR conditions were as follows: denaturation at 94°C for 5 min, followed by 35 cycles of denaturation at 94°C for 30 sec, annealing at 55°C for 30 sec, extension at 72°C for 30 sec and ending with an extension at 72°C for 10 min. The PCR products were sequenced bidirectionally using the ABI 3730xl DNA analyzer (Applied Biosystems, Bedford, MA, USA).

### RNA preparation and real-time quantitative PCR

The total RNA was extracted using TRIzol (Invitrogen Life Technologies, Carlsbad, CA, USA), and the cDNA was prepared using M-MLV reverse transcriptase (Promega Corporation, Madison, WI, USA) according to the manufacturer’s instructions. Reverse transcription was performed at 37°C for 15 min, followed by 85°C for 5 sec. Real-time quantitative PCR (RQ-PCR) was performed using the ABI Prism 7500 system (Applied Biosystems) according to the manufacturer’s instructions. PCR was performed in a total volume of 10 *μ*l, which included 5 *μ*l 2X SYBR Green real-time PCR master mix (Toyobo Co. Ltd., Osaka, Japan), PCR-grade water, 1 *μ*l template cDNA and 0.5 *μ*l forward and reverse primers. The sequences of the target-specific primers were designed from human cDNA sequences available in GenBank. DNMT3A forward, 5′-GCC ACC TCT TCG CTC CGC TG-3′ and reverse, 5′-GAT GAT GTC CAA CCC TTT TCG CAA-3′; and β-actin forward, 5′-TGA CGT GGA CAT CCG CAA AG-3′ and reverse, 5′-CTG GAA GGT GGA CAG CGA GG-3′. The thermal cycling profile consisted of 95°C denaturation for 5 min, followed by 40 cycles at 95°C for 15 sec, 65°C for 15 sec and 72°C for 45 sec. To exclude non-specific amplification and primer-dimer formation, a dissociation curve analysis was performed and PCR products were confirmed by agarose gel electrophoresis. PCR-grade water was used instead of template cDNA for the negative control. The fold-change in the gene expression was determined using the 2^−ΔCT^ method with β-actin as an endogenous control. All experiments were performed at least twice.

### Statistical analysis

The Student’s t-test was used to compare the differences in DNMT3A expression levels between the AML patients with R882 mutations and the normal controls. The difference in the DNMT3A expression levels between M5 subtype AML and the other AML subtypes was also compared by Student’s t-test. Differences in the DNMT3A expression levels were compared by an analysis of variance in three groups. The clinical characteristics of the AML and ALL patients, including gender, age, white cell count and other factors, are presented in Tables I and II. The AML and ALL patients were categorized into high and low DNMT3A-expressing subgroups using the median value as the cut-off. Fisher’s exact test was used to compare the CR rate in patients with an intermediate-risk profile. Pearson’s chi-square test was used to compare the CR rates between other groups. P<0.05 was considered to indicate a statistically significant difference. Statistical analysis was performed using the SPSS 17.0 statistical software program (SPSS Inc., Chicago, IL, USA).

## Results

### DNMT3A mutations

**Figure 1.**
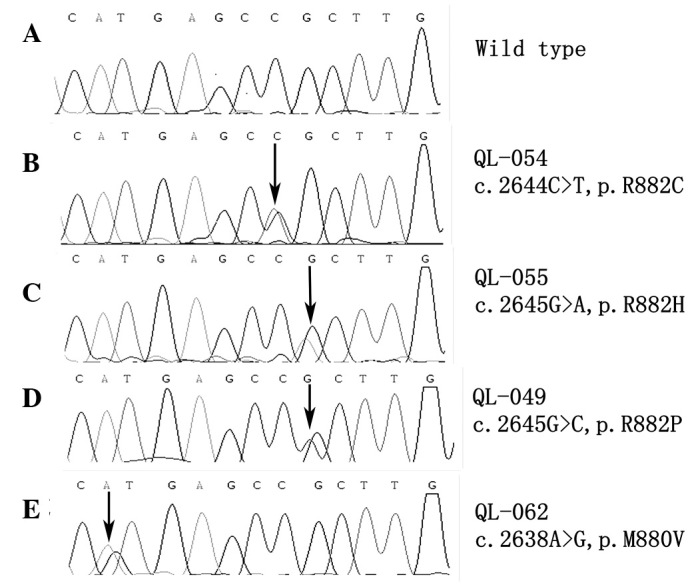
Sequencing of DNMT3A mutations in patients with AML. (A) Wild-type and (B–E) mutated DNMT3A gene Arrow indicates the mutated site. Three heterozygous mutations were identified at codon 882 in the samples of nine AML patients, and the sequencing figures of three patients are presented. (E) A novel SNP causing amino acid substitution, M880V, was found in a female patient with AML. The unique patient number (UPN) and mutation details of these patients are provided to the right of the peak charts. DNMT3A, DNA methyltransferase 3A; AML, acute myeloid leukemia; SNP, single nucleotide polymorphism.

DNMT3A R882 mutational status was determined in a cohort of 57 AML and 41 ALL patients and 1 BAL patient. Of the AML patients, 6 were identified to exhibit the R882H variant, two the R882C variant, one the R882P variant and one the M880V variant, a novel single nucleotide polymorphism that leads to amino acid substitution. The DNMT3A mutation frequency in AML was 17.5% (10/57). Sequencing results of each type of mutation are presented in Fig. 1. None of the DNMT3A mutations were found in the ALL and BAL patients. The AML patients with DNMT3A mutations revealed lower CR rates following induction therapy compared with those with wild-type DNMT3A (0 vs. 62.8%; P<0.001). The presence of a DNMT3A mutation was found to correlate with a low CR rate in the AML patients with an intermediate-risk profile (P=0.061).

### Clinical features of patients with DNMT3A mutations

The association between the status of DNMT3A mutations and clinical features in AML was investigated. Patients with DNMT3A mutations were classified with M5 subtype AML. The age and white cell count of the AML patients with DNMT3A mutations were higher than those without DNMT3A mutations. Gender and percentage of bone marrow blasts at diagnosis were not found to be significantly different between the two groups. DNMT3A mutations were significantly enriched in 8/26 patients with a cytogenetic profile associated with intermediate risk (30.8%; P=0.004; Table I). The clinical and genetic characteristics of the 10 DNMT3A-mutated AML cases are presented in Table III.

### DNMT3A expression level

**Figure 2.**
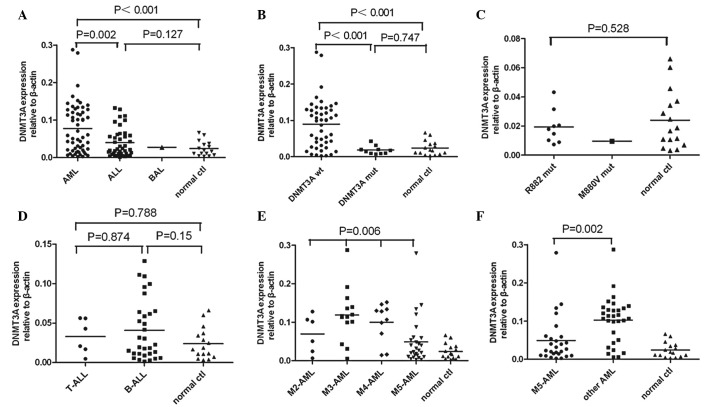
DNMT3A expression levels. Each dot represents one sample. (A) DNMT3A expression for 41 ALL patients, 57 AML patients, 1 BAL patient and the normal controls (n=16). DNMT3A expression in AML patients with (B) wild-type or (C) R882 or other DNMT3A mutations compared with normal controls. (D) 37 ALL patients are divided into 6 T-ALL and 31 B-ALL patients. No difference was identified between DNMT3A expression levels in T-ALL and B-ALL patients. (E) DNMT3A expression levels for various AML subtypes according to FAB classification. (F) DNMT3A expression levels for M5 subtype AML were lower compared with other types of AML. DNMT3A, DNA methyltransferase 3A; ALL, acute lymphoblastic leukemia; AML, acute myeloid leukemia; BAL, biphenotypic acute leukemia; T, T-cell; B, B-cell; FAB, French-American-British.

The DNMT3A expression levels were measured using RQ-PCR. DNMT3A expression in the AML patients was found to be significantly higher than that of the ALL patients or normal controls (P=0.002 or P<0.001). DNMT3A expression was significantly decreased in the AML patients with DNMT3A mutations, including R882 and M880 mutations, compared with individuals without mutations (P<0.001). The AML patients with wild-type DNMT3A revealed significantly higher DNMT3A expression levels compared with the normal controls (P<0.001). No statistical difference was identified between the AML patients with DNMT3A mutations and the normal controls (P=0.747). The ALL patients demonstrated higher DNMT3A expression levels compared with the normal controls, however, this difference was not statistically significant (P=0.127). No difference was found in DNMT3A expression between the T-cell ALL and B-cell ALL patients (P=0.874). DNMT3A expression between the different AML subtypes (P= 0.006) was significantly different. The M5 subtype AML patients were found to exhibit significantly lower DNMT3A expression levels compared with the patients with other subtypes of AML, including the M2, M3 and M4 subtypes (P=0.002; Fig. 2).

To determine whether DNMT3A expression levels affect the treatment response of AML patients, the patients were divided into 2 groups; those with low or high DNMT3A expression (below or above the median level, respectively). The CR rate was calculated for each group according to the DNMT3A expression levels. The group with low DNMT3A expression revealed a lower CR rate than that of the high DNMT3A expression group (30.8 vs. 70.4%; P= 0.002). The ALL patients were also divided into two groups using the same method. No significant difference was observed in the CR rate (57.9 vs. 60%; P=0.894) between the two groups or between T-cell and B-cell lymphoblastic leukemia (33.3 vs. 62.1%; P= 0.195).

## Discussion

To date, DNMT3A mutations have been detected in AML, CML, CMML, MDS, lymphoma and MPN. The frequency of DNMT3A mutations in AML is the highest when compared with other heterogeneous malignancies. Recently, Ribeiro *et al* reported that mutant DNMT3A represents an independent prognostic marker in AML. When patients with DNMT3A mutations at position R882 were analyzed, an association with an inferior outcome was also observed (16). In the present study, 10 mutations were identified in DNMT3A in 10/57 (17.5%) *de novo* AML patients. This high frequency is consistent with results of previous studies on DNMT3A mutations in AML patients (5,17). In addition, the mutation of DNMT3A was found to correlate with a low CR rate in AML patients with an intermediate-risk profile, indicating that the mutation of DNMT3A represents a novel prognostic index for intermediate-risk AML patients. However, none of the DNMT3A R882 mutations were identified in this consecutive series of ALL cases. Prior to the present study, Kim *et al* reported that the frequency of DNMT3A mutations in adult ALL was extremely low (0.8%, 1/124) (18). Differences in the frequency of DNMT3A mutations between AML and ALL may be associated with the different pathogenic mechanisms in AML and ALL. This hypothesis must be studied further, using larger cohorts to identify DNMT3A mutations in ALL patients and to evaluate the prognostic impact of the mutations.

In the present study, DNMT3A R882 mutations were observed to be recurrent in AML patients and associated with a poor clinical outcome. DNMT3A is markedly over-expressed in the majority of AML patients with wild-type DNMT3A when compared with normal controls. However, no difference was identified in DNMT3A expression between the AML patients with DNMT3A mutations and the control individuals. These results indicate that DNMT3A mutations may reduce the methyltransferase activity. Therefore, we hypothesized that the reduced expression of DNMT3A is indicative of a poor clinical outcome in AML patients due to decreased methyltransferase activity. This hypothesis is consistent with the observations that M880V and all R882 mutations are heterozygous and that this mutation reduces methyltransferase activity *in vitro* (19). Therefore, DNMT3A expression may represent a potential biomarker for the prediction of prognosis in AML.

In the present study, the DNMT3A expression levels in AML patients were compared between various FAB subtypes, and a significant difference was identified. DNMT3A expression in M5 subtype AML was lower than that of other AML subtypes. In addition, the frequency of DNMT3A mutations in the AML patients with the M5 subtype was higher compared with the patients of other subtypes, indicating that decreased DNMT3A expression caused by DNMT3A mutations may be associated with the incidence and progression of AML, particularly in the M5 subtype.

The difference in the CR rate between the two groups of AML patients indicated that lower DNMT3A expression correlated with an adverse treatment response. In the ALL patients, no difference was found in the CR rate, which may be explained by the marked difference in the DNMT3A mutation status and expression levels between AML and ALL. These results indicate that the function of DNMT3A in gene methylation in ALL may be distinct from its role in AML.

A number of studies have confirmed that the DNA methylation of specific genes is associated with the clinical outcome (3,20,21) and that the activity of DNA methyltransferases may contribute to specific DNA methylation profiles. Previously, Challen *et al* analyzed the effect of hematopoietic-specific conditional Dnmt3a deletion on self-renewal in serial transplantation assays (14). Using conditional ablation, the study reported that Dnmt3a loss progressively impaired mouse hematopoietic stem cell (HSC) differentiation. Dnmt3a-null HSCs were found to exhibit increased and decreased methylation at distinct loci, including substantial CpG island hyper- and hypomethylation. In the Dnmt3a-null HSCs, an extremely large number of hypomethylated genes were found that are commonly overexpressed in different types of leukemia, including AML and ALL. These observations are indicative of a crucial role for Dnmt3a in the pathogenesis of malignant neoplasms. However, the Dnmt3a-null status in HSCs in mice is distinct from DNMT3A mutations in humans, as all R882 mutations are heterozygous. Whether the same set of genes is subjected to altered epigenetic patterning in DNMT3A-mutant AML cells has not been investigated.

TET2 and NPM1 mutations are markedly associated with DNMT3A mutations in T-cell lymphoma and adult AML, respectively (5,10,17). These studies indicate an oncogenic cooperation between DNMT3A and other gene mutations, resulting in the deregulation of the cytosine methylation and demethylation processes. In the present study, the patients with DNMT3A mutations were older than the patients without DNMT3A mutations in AML, which was consistent with the results obtained by Ley *et al* (5). Consistent with these observations, a low frequency of DNMT3A mutations in pediatric AML was observed in studies by Ho *et al* (0/180, 0%) (22) and Hollink *et al* (3/140, 2.1%) (23). In pediatric AML, there is a 4–5-fold lower frequency of NPM1 mutations compared with adult AML (24). These results may partially explain why the frequency of DNMT3A mutations is low. Therefore, we hypothesized that DNMT3A mutations alone are insufficient to generate AML and other malignancies, and that second hits may be required.

The results of the present study, in combination with observations of previous studies, indicate that DNMT3A mutations are associated with adverse outcomes in AML and that they may represent a novel marker for the risk stratification of AML. By contrast, DNMT3A mutations in ALL are rare. At present, the mechanisms by which mutated DNMT3A regulates DNA methylation remain unclear. Additional studies must be performed to identify and understand the regulatory mechanisms of DNMT3A. Screening for DNMT3A mutations may provide a novel tool for the prediction of clinical outcome.

## Figures and Tables

**Table I.** Clinical characteristics of 57 patients with AML.

Characteristics	No DNMT3A mutation	R882 mutation	Non-R882 mutation	Any DNMT3A mutation	P-value^a^
Patients, n	47	9	1	10	
Age at study entry, years	42.4±16.1^b^	60.3±16.5^b^	55	59.8±15.7^b^	0.007^c^
Male gender (%)	26 (55.3)	3 (33.3)	0 (0.0)	3 (30.0)	0.179^d^
Bone marrow blasts at diagnosis, %	77.75±20.84^b^	84.9±19.7^b^	87	84.9±19.7^b^	0.073^c^
Normal karyotype, n/total (%)	11/13 (84.6)	4/4 (100)	1/1 (100.0)	5/5 (100.0)	
White-cell count at diagnosis, ×10^3^ cells/mm^3^					
Mean	26.71±45.57^b^	114.28±88.34^b^	22.38	105.09±88.21^b^	<0.001^b^
Median	10.93	103.00	22.38	94.47	
Cytogenetic risk, n/total (%)					0.004^d^
Favorable	18/45 (40.0)	0/7 (0.0)	0/1 (0.0)	0/8 (0.0)	
Intermediate	19/45 (42.2)	7/7 (100.0)	1/1 (100.0)	8/8 (100.0)	
Adverse	8/45 (17.8)	0/7 (0.0)	0/1 (0.0)	0/8 (0.0)	
AML subtype, n (%)					
M2	6 (12.8)	0 (0.0)	0 (0.0)	0 (0.0)	
M3	14 (29.8)	0 (0.0)	0 (0.0)	0 (0.0)	
M4	10 (21.3)	0 (0.0)	0 (0.0)	0 (0.0)	
M5	17 (36.2)	9 (100.0)	1 (100.0)	10 (100.0)	

a P-values, no DNMT3A mutations vs. any DNMT3A mutation.

b Data are presented as mean ± SD. P-values were calculated using

c Fisher’s exact or

d Wilcoxon tests. AML, acute myeloid leukemia.

**Table II.** Clinical characteristics of 41 ALL patients and one BAL patient.

Characteristics	T-cell leukemia	B-cell leukemia	ALL with unknown phenotype	BAL
Patients, n	6	31	4	1
DNMT3A mutations, n (%)	0 (0.0)	0 (0.0)	0 (0.0)	0 (0.0)
Age at study entry, years	28.3±24.7^a^	37.4±16.7^a^	43.0±24.6^a^	60
Male gender, n (%)	4 (66.7)	16 (51.6)	2 (50.0)	1 (100.0)
Bone marrow blasts at diagnosis, %	95.0±0.0^a^	86.1±13.8^a^	83.5±12.0^a^	97
Normal karyotype, n/total (%)	4/4 (100.0)	9/23 (39.1)	0/2 (0.0)	0/0 (0.0)
White cell count at diagnosis, ×10^3^ cells/mm^3^				
Mean	52.1±67.8^a^	63.0±98.4^a^	2.54±1.94^a^	5.08
Median	16.26	11.17	2.33	5.08
Aberrant karyotype, n/total (%)				
(9,22)(q34;q11) or BCR/ABL fusion gene	0/4 (0.0)	13/23 (41.9)	0/2 (0.0)	0/0 (0.0)

a Data are presented as mean ± SD. ALL, acute lymphoblastic leukemia; BAL, biphenotypic acute leukemia.

**Table III.** Clinical and genetic characteristics of the ten DNMT3A-mutated AML cases.

UPN	Nucleotide change	Consequence	Age, years	Gender	FAB	Karyotype	Aberrant expression or mutation of other genes	Response or outcome following induction chemotherapy
QL-049	c.2645G>C	p.R882P	37	Female	M5	46, XX	None	PR
QL-053	c.2645G>A	p.R882H	79	Male	M5	46, XY	None	Deceased
QL-054	c.2644C>T	p.R882C	44	Male	M5	46, XY	None	NR
QL-055	c.2645G>A	p.R882H	41	Female	M5	Unknown	WT1 (+)	NR
QL-056	c.2644C>T	p.R882C	65	Male	M5	46, XY	None	NR
QL-058	c.2645G>A	p.R882H	71	Female	M5	Unknown	None	NR
QL-062	c.2638A>G	p.M880V	55	Female	M5	46, XX	WT1 (+)	NR
QL-067	c.2645G>A	p.R882H	60	Female	M5	46, XX	WT1 (+)	NR
QL-084	c.2645G>A	p.R882H	63	Female	M5	46, XX	NPM1 (+), CEBPA (+)	NR
QL-092	c.2645G>A	p.R882H	83	Female	M5	46, XX	FLT3 (+)	Deceased

UPN, unique patient number; FAB, French-American-British; PR, partial remission; NR, non-remission.
